# Metabolic profiles of Cuibi-1 and Zhongyan-100 flue-cured tobacco leaves in different growing regions by gas chromatography/mass spectrometry

**DOI:** 10.1098/rsos.180261

**Published:** 2018-05-30

**Authors:** Bo Sun, Ai-Hong Zheng, Fen Zhang, Ke-Su Wei, Qing Chen, Ya Luo, Yong Zhang, Xiao-Rong Wang, Fu-Cheng Lin, Jun Yang, Hao-Ru Tang

**Affiliations:** 1 College of Horticulture, Sichuan Agricultural University, Chengdu 611130, People's Republic of China; 2 Zhengzhou Tobacco Research Institute, Zhengzhou 450001, People's Republic of China; 3 Guizhou Academy of Tobacco Science, Guiyang 550081, People's Republic of China

**Keywords:** metabolic profiles, GC–MS, flue-cured tobacco leaves, growing regions, cultivars

## Abstract

The metabolic profiles of tobacco leaves of two differential Chinese cultivars from different growing regions were analysed using gas chromatography–mass spectrometry (GC–MS). The results of principal component analysis, partial least-squares discriminant analysis and hierarchical cluster analysis showed significant differences in metabolome among three groups, identified 24 differential metabolites, and analysed the metabolic pathway in which the metabolites were involved. Among them, 13 metabolites were associated with geographical regions, including seven organic and fatty acids, four carbohydrates and two secondary metabolites. Four amino acids and two monosaccharides were associated with cultivars and the remaining five metabolites were associated with both. The relationships among the differential metabolites and the distinct characteristics of environment and cultivar were further discussed. In addition, correlation analysis indicated that most of the differential carbohydrates were negatively correlated with the differential amino acids and organic acids. Taken together, this study demonstrates the metabolite differences between two cultivars in different regions, and highlights the effect of environment and cultivar on tobacco leaf metabolism.

## 1. Introduction

Plant metabolomics is a comprehensive method used to estimate global metabolite changes [1–3], and has been applied in several plant research fields, such as biomarker selection [4,5], gene function research [6], assessment of genetically modified plants [7], identification of differential metabolites and pathways among different cultivars [8–10], developmental stages [11] and environments [1,2]. The common analytical platforms in plant metabolomics studies are gas chromatography–mass spectrometry (GC–MS), liquid chromatography–mass spectrometry (LC–MS), capillary electrophoresis–mass spectrometry (CE–MS) and nuclear magnetic resonance (NMR) [12]. Today, GC–MS has become one of the most applicable methods in plant metabolomics, and has good separation efficiency for polar small molecular metabolites and identification via various mass spectral libraries [12].

Numerous studies have shown that growing regions and climatic conditions have a significant impact on metabolites in tobacco leaves. Flue-cured tobacco leaves grown in China are divided into clear flavour, full flavour and middle flavour types, and tobacco leaves from Yunnan, Henan and Guizhou Provinces have the typical characteristics of these three above flavour types, respectively [13]. The remarkable metabolite differences in tobacco leaves from the three geographical origins have been analysed by using GC–MS, LC–MS and CE–MS alone or in combination, and differential metabolites were also identified [1,2,14–16]. Hubei Province is one of the important planting areas for Chinese tobacco, and is located in central China, between Guizhou Province and Henan Province. Similar to tobacco leaves from Guizhou Province, leaves from Hubei Province are also classified as middle flavour type [13]. However, little is known about the metabolic profile of flue-cured tobacco leaves from Hubei Province. Therefore, we chose Hubei and Yunnan as the growing origins in our study.

In addition to the geographical origin factor, the quality and metabolic profiles of tobacco leaves are also obviously influenced by the cultivar factor. Tobacco is an important cash crop distributed widely in China, and lots of flue-cured tobacco cultivars have been bred to adapt to various geographical growing locations with great environmental differences [17]. The chemical components, such as alkaloids and aroma substances, were diffusely analysed and compared among different tobacco cultivars, and significant differences were observed [18–21]. In our study, two special and differential Chinese cultivars were selected and analysed. Cuibi-1, which was selected from the Sanming tobacco planting area in Fujian Province, has an elegant, graceful and pure aroma, and is susceptible to black shank and bacterial wilt infections [22]. Zhongyan-100 is a cultivar that is highly resistant to brown spot and black shank, and has favourable chemical composition and good adaptability [23]. In this study, we analysed the metabolic profile of these two Chinese tobacco middle leaves in different regions using GC–MS, and identified their differential metabolites via a series of multivariate analyses. Their involved metabolic pathways and correlation of differential metabolites were also analysed.

## 2. Experimental

### 2.1. Plant materials

Two Chinese commercial flue-cure tobacco cultivars (Cuibi-1 and Zhongyan-100) were chosen and used in this study. Cuibi-1 cultivars were planted in Yuxi City, Yunnan Province (YXC) and Xuanen County, Hubei Province (XNC), respectively, while Zhongyan-100, which is unsuitable growing in Yunnan due to climatic reasons, was only planted in Xuanen County (XNZ) (table 1). The annual average temperature, annual precipitation, annual sun exposure time and altitude of the planting region in Xuanen County were 15.8°C, 1490 mm, 1136 h and 1000 m.a.s.l., respectively, while those in Yuxi City were 16°C, 900 mm, 2200 h and 1800 m.s.l., respectively. The data were obtained from China Meteorological Administration. Seeds were germinated and planted in the greenhouse. Then 100 seedlings of each cultivar with 7–9 true leaves were transplanted into an agricultural field with 120 cm between rows and 60 cm between plants within rows. The field design was completely random. Water, fertilizer and pesticides were applied, as and when required.

**Table 1.** The information for plant material sampling.

index	cultivar	growing site	sampling leaf position	sampling date
XNC	Cuibi-1	Xuanen County, Hubei Province, China	middle part (11–13)	12 August 2012
YXC	Cuibi-1	Yuxi City, Yunnan Province, China	middle part (11–13)	1 August 2012
XNZ	Zhongyan-100	Xuanen County, Hubei Province, China	middle part (11–13)	7 August 2012

Plant materials without any insects and mechanical damage were sampled from middle leaf positions (no. 11–13) at the mature leaf stage. Leaves from five tobacco plants were collected as a replicate, and six independent replicates were taken for analysis. After harvest, the leaf samples were immediately frozen in liquid nitrogen, lyophilized to dryness, and ground to a fine powder for subsequent analyses in a laboratory of the Zhengzhou Tobacco Research Institute.

### 2.2. Sample preparation

Metabolites were extracted from lyophilized tobacco samples and analysed as previously described with minor modification [1]. A 20 mg sample of leaf powder was added to 1.0 ml of extraction solution, which consisted of methanol, chloroform and ddH_2_O with a ratio of 5/2/2 (v/v/v), and 0.2 ml of ribitol (40 µg ml^–1^) as the internal standard, respectively. The solvent mixture was extracted by an ultrasonic method for 40 min at room temperature. After vortexing for 30 s, the extraction solution was centrifuged at 12 000 r.p.m. for 20 min at room temperature. Subsequently, 400 µl of the supernatant was transferred to a 2 ml Eppendorf tube and dried by nitrogen evaporator. For derivatization, 50 µl of methoxyamine hydrochloride dissolved in pyridine (20 mg ml^–1^) was added, and vortexed for 1 min, and then incubated for 90 min at 37°C. For silylation, 80 µl of *N*-methyl-*N*-(trimethylsilyl)trifluoroacetamide (MSTFA) was added, and incubated for 30 min at 37°C, and then vortexed for 30 s. The solution was left standing for 1 h, and centrifuged at 12 000 r.p.m. for 10 min, and then 80 µl of the supernatant was transferred to a trace sample vial for further analysis.

### 2.3. Gas chromatography–mass spectrometry analysis

GC–MS analysis of the metabolites in the tobacco leaves was carried out using an Agilent 7890A gas chromatograph (GC) interfaced to an Agilent 5975C mass-selective detector (Agilent, USA), controlled by an Agilent G1701EA GC-MSD ChemStation. Samples were tested in random order with quality control (QC) samples being inserted with every six samples in the running sequence. QC samples were mixed with the same amount of each sample. Chromatographic separations were achieved in an DB-5 (30 m × 0.250 mm, 0.25 µm film thickness) capillary column (Agilent Technologies Inc., USA). The column flow, using helium as the carrier gas, was held constant at 1.0 ml min^−1^. The column temperature was set to 70°C for 4 min, and programmed to 310°C at 5°C min^−1^ and kept at this temperature for 10 min. The injector temperature was 290°C, and the injector was set in split mode (10 : 1) with an injection volume of 1 μl. The interface temperature was 230°C and the ion source temperature was 280°C. Mass spectra were recorded at 70 eV, and used both full scan and selected ion monitoring (SIM) mode with scanning from 40 to 510 amu. Ions were acquired with a solvent cut time of 8.0 min [1].

### 2.4. Data processing and statistical analysis

Quantitative analysis, which used a pseudo-targeted method, was carried out in SIM mode [12,24]. The components eluting within the total ion chromatogram were extracted in the Agilent MSD ChemStation. Then the metabolites were identified in two principal ways: (1) the identification of metabolites was based on mass spectral matching with NIST and Fiehn mass spectra libraries and (2) 49 commercial standards were used to confirm unambiguously (electronic supplementary material, table S1). Subsequently, the peaks detected in less than 80% of the samples were discarded according to the ‘80% rule' [1], and the peak area of metabolites was normalized to the internal standard for further data analysis. Principal component analysis (PCA) was performed by Simca-p 13.0 Demo software (Umetrics, Sweden) with Pareto scaling to understand the relationships among samples. To investigate the differences in metabolite levels among tobacco leaf samples, partial least-squares discriminant analysis (PLS-DA) was then carried out by Simca-p 13.0 Demo software with Pareto scaling [1]. These metabolites, which had variable importance in the projection (VIP) value > 1 (PLS-DA) and *p* < 0.05 (Mann–Whitney *U* test by SPSS18.0 software), were identified as significant differential metabolites [2]. TIGR MeV software (Version 4.1) was used to cluster the significant differences in metabolite levels [20]. Metabolic pathway enrichment analysis was performed to confirm the important pathways related to metabolic phenotype according to the KEGG website (www.genome.jp/kegg/). The correlation between differential metabolites was visualized using Cytoscape v. 3.5.1 software.

## 3. Results

### 3.1. Pseudo-targeted gas chromatography–mass spectrometry selected ion monitoring analysis

**Figure 1.**
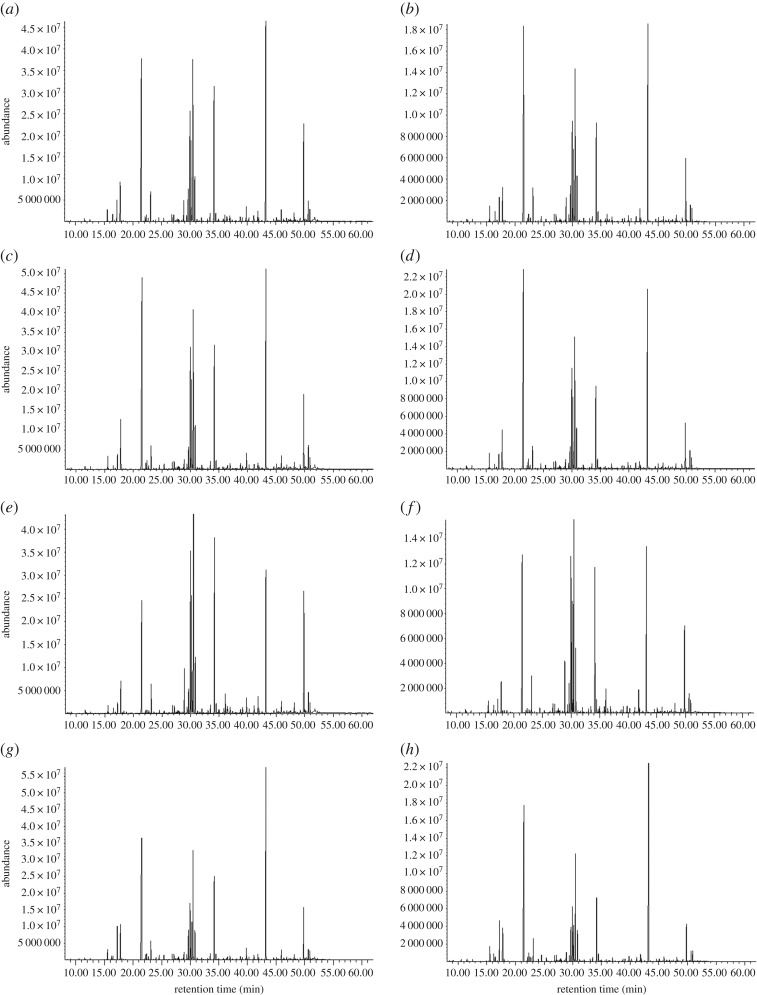
GC–MS total ion chromatogram (TIC) of tobacco samples. (*a,b*) QC samples; (*c,d*) XNC samples; (*e*) and (*f*) YXC samples; (*g*,*h*) XNZ samples. (*a,c,e,g*) Were analysed in full scan mode, whereas (*b,d,f,h*) were analysed in SIM mode.

To establish the pseudo-targeted GC–MS SIM method, a QC sample was analysed with full scan mode, and the Agilent MSD ChemStation was used to output the raw data. The distinction of overlapped peaks and selection of characteristic ions for metabolites were performed by Automatic Mass Spectral Deconvolution and Identification System (AMDIS) software. The data were divided into 27 groups based on retention time (RT) and characteristic ions. The total ion chromatograms (TICs) from the full scan and pseudo-targeted SIM mode of the QC sample is shown in figure 1. The pseudo-targeted GC–MS SIM method established with 27 groups and 313 quantitative ions was used to investigate the metabolic difference between fresh flue-cured tobacco middle leaves from different cultivars and different geographical origins.

The compound identification was performed based on matching with mass spectra libraries and commercial standards. Among the 313 peaks, 87 compounds were identified totally, and 49 compounds were confirmed by standards (electronic supplementary material, table S1). Among the identified compounds, the primary metabolites, such as amino acids, sugars, and organic and fatty acids, were abundant in samples. Some kinds of secondary metabolites influencing tobacco leaf quality, such as alkaloids, chlorogenic acid and sterols were also detected.

**Figure 2.**
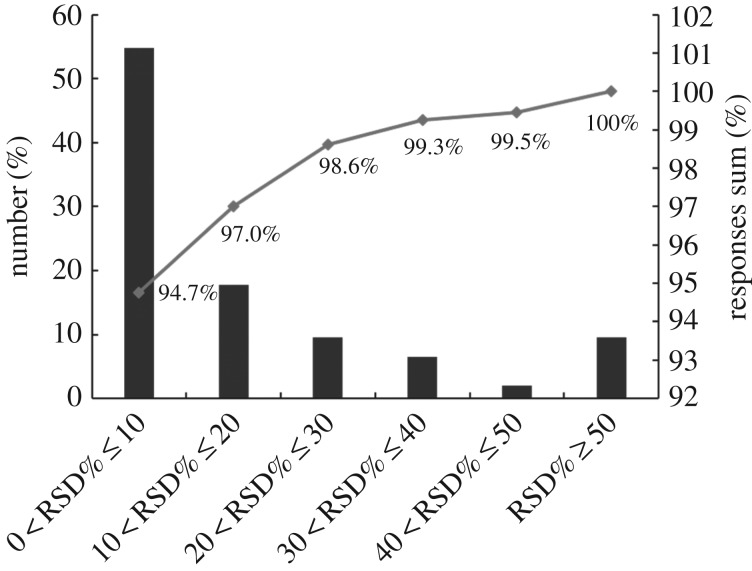
RSD distribution for all metabolites in three QC samples. The black columns represent the percentage of the peak number within the specified RSD% range, respectively. The line indicates the cumulative percentage of the peak area within the specified RSD% range.

To inspect the reproducibility of the pseudo-targeted SIM method, relative standard deviation (RSD) of all 313 peaks were calculated based on their peak area in the QC samples. The results showed that 82% of the peaks, which were up to 98.62% of the total peak area, had an RSD of less than 30% (figure 2), indicating that this method is reproducible and could be used in the subsequent experiment. It is hard to tell the differences among fresh tobacco leaf samples from different cultivars and geographical origins based on chromatograms (figure 1), so a multivariate statistical analysis was important for further data excavation.

### 3.2. Principal component analysis

**Figure 3.**
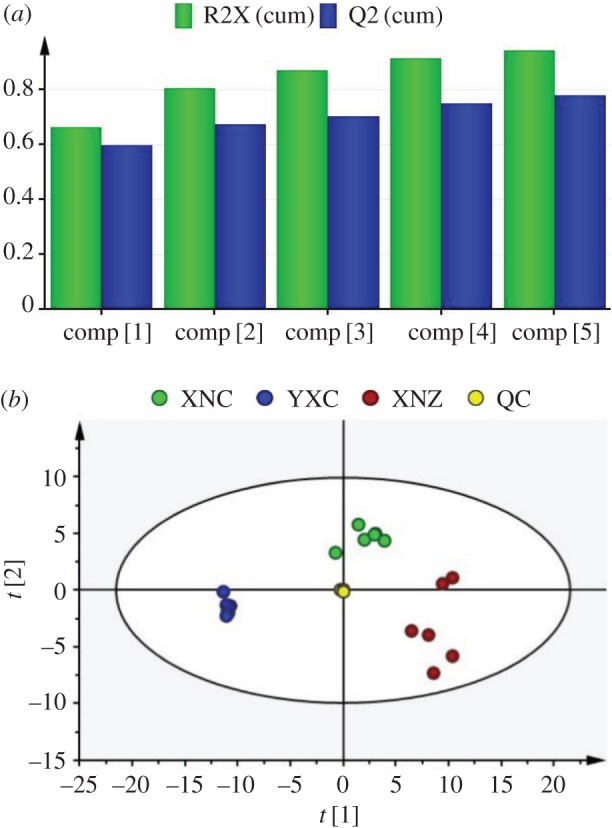
Summary of the fit (*a*) and score plot (*b*) of PCA of tobacco samples from different cultivars and geographical origins. Green and blue columns in (*a*) represent the cumulative R2X and Q2, respectively. Yellow, red, green, blue circles in (*b*) represent the QC, XNZ, XNC, YXC samples, respectively.

To get an overview of the effect of cultivar and growing site on tobacco leaf metabolomes, the metabolic data for Cuibi-1 and Zhongyan-100 middle leaves from two locations (Xuanen County and Yuxi City) were analysed using PCA analysis, which is an unsupervised multivariate analysis method. As shown in figure 3*a*, five principal components of the PCA plot describe 94.4% of the total variance information, while the first principal component is as high as 66.6%. Moreover, all of the QC samples were clustered together and located at the centre of the PCA score plot (figure 3*b*), indicating good analytical stability and reproducibility of this experiment. In the PCA model, three groups of tobacco leaf samples were well separated along the first principal component, and XNC samples were dispersed between the YXC and XNZ samples (figure 3*b*), indicating that both cultivar and growing site had great impact on the metabolic profile of tobacco leaves.

### 3.3. Partial least-squares discriminant analysis

**Figure 4.**
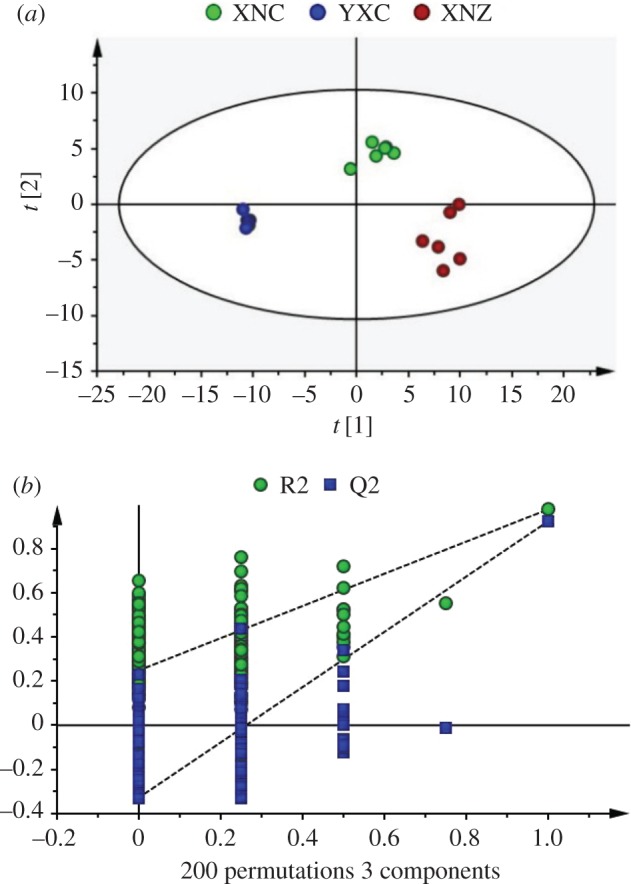
Score plot (*a*) and cross-validation plot (*b*) of the PLS-DA of tobacco samples from different cultivars and geographical origins. Red, green, blue circles in (*a*) represent the XNZ, XNC, YXC samples, respectively. Three components were fit by autofit model, and the parameters of the model were as follows: R2Y(cum) = 0.982, Q2(cum) = 0.953. (*b*) Cross-validation plot of PLS-DA mode with 200 permutation tests. The intercepts of R2 and Q2 were 0.249 and −0.327, respectively.

To further investigate the differences among tobacco leaf samples, a PLS-DA model was established (figure 4). Three groups of tobacco leaf samples were clearly separated from the score plot (figure 4*a*), and the distribution trend is similar to the PCA results. In this PLS-DA model, three components described 98.2% of the variation and predicted 95.3% according to cross-validation; R2Y and Q2 of the first two components were predominant among them, and the values were 92.3% and 89.0%, respectively. The results showed that the PLS-DA model had good robustness properties, and the selection of the first two components was large enough to examine this dataset. A permutation test could be used to evaluate the possible overfitting of the PLS-DA model, and a properly fitted model was identified as having values of R2-intercept less than 0.4 and a Q2-intercept less than 0.05 [25]. In this study, 200 permutation tests were performed; the R2-intercept was 0.249 and Q2-intercept was −0.327 (figure 4*b*), indicating that the PLS-DA model had no overfitting and was credible.

**Figure 5.**
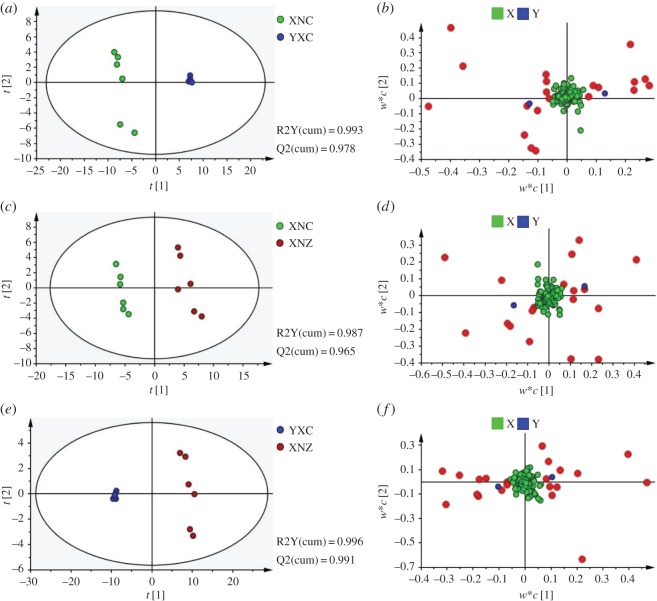
PLS-DA analysis of tobacco samples from different cultivars and geographical origins. (*a*) Score plot of XNC and YXC. (*b*) Loading plot of XNC and YXC. (*c*) Score plot of XNC and XNZ. (*d*) Loading plot of XNC and XNZ. (*e*) Score plot of YXC and XNZ. (*f*) Loading plot of YXC and XNZ. Red circles in loading plots represent the compounds whose VIP value was more than 1.

**Figure 6.**
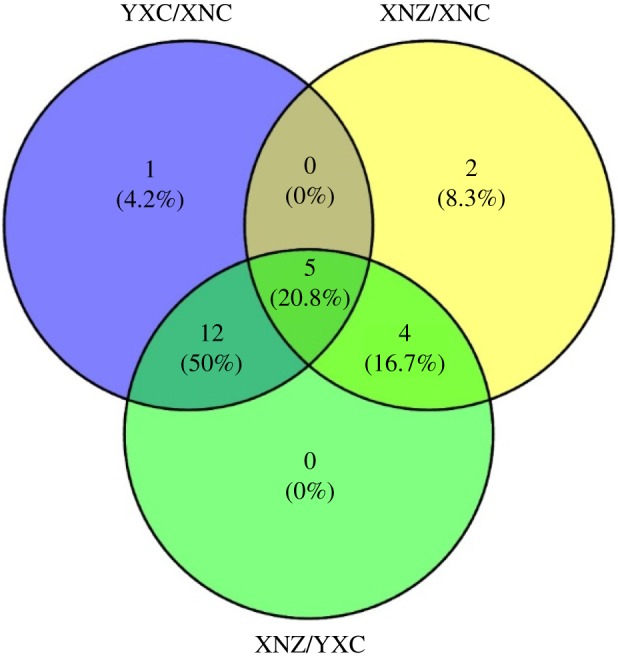
Venn diagram of the differential metabolites in a pair of samples.

The identification of differential metabolites between each two samples was performed using VIP values and further confirmed by the non-parametric Mann–Whitney *U* test. Among the three groups, a total of 27 differential metabolites with VIP greater than 1 and *p* < 0.05 were screened out (figure 5). Of these, 24 metabolites were verified via commercial standards and mass spectra libraries. A Venn plot showed that 18 metabolites in YXC samples and 11 metabolites in XNZ samples were significantly different from XNC samples, and 21 differential metabolites were observed between YXC samples and XNZ samples (figure 6). In addition, five metabolites, sucrose, fructose, cellobiose, inositol and propanoic acid, were significantly different among the three pairwise comparisons. The relative concentration, VIP value, *p*-value and fold change of differential metabolites are shown in supporting information in electronic supplementary material, table S2.

### 3.4. Hierarchical cluster analysis

**Figure 7.**
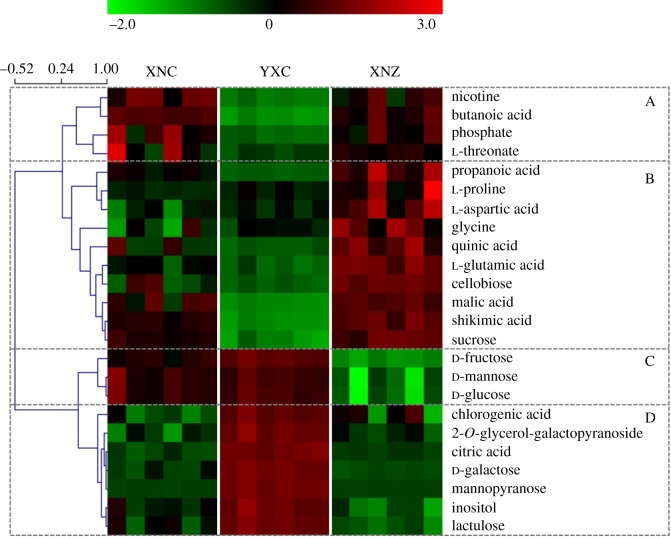
Heat map of the differential metabolites. Green and red reflect the relative concentration of the metabolites.

The hierarchical cluster analysis (HCA) of identified metabolites was performed to visualize the clustering of 24 differential compounds (figure 7). To determine the relationships and trends of the differential metabolites among the three groups of samples, the heat map was divided into four groups. The metabolites in group A had the highest number of the XNC samples and the lowest number of YXC samples. Significant differences in metabolite levels were observed between the YXC samples and two other samples, whereas no significant difference was found between the XNC samples and the XNZ samples. Group B is the largest group in figure 7, and is shown as a cluster of 10 differential metabolites, including four amino acids (proline, aspartic acid, glycine and glutamic acid), four organic acids (propanoic acid, malic acid, quinic acid and shikimic acid), and two disaccharides (cellobiose and sucrose). The levels of all the metabolites in group B were the highest in the XNZ samples, and most of these were significantly different from the two other samples, except for quinic acid and malic acid. Different trends of the metabolites in group B were observed between the XNC samples and the YXC samples. The levels of three amino acids (proline, aspartic acid and glycine) in the XNC samples were less than those in the YXC samples, but there were no notable differences between them. However, the levels of other metabolites in group B in the XNC samples were significantly higher than those in the YXC samples (electronic supplementary material, table S2). In group C, the metabolites included three monosaccharides (fructose, mannose and glucose), and their levels were the highest in the YXC samples and lowest in the XNZ samples, while significant differences were found between the XNZ samples and two other samples. Group D mainly contained some sugars (e.g. galactose, inositol, and lactulose), organic acid (citric acid) and phenolics (chlorogenic acid). The concentrations of the corresponding metabolites in group D were significantly higher in the YXC samples, and no significant differences were found between two other samples grown in Xuanen County, except for inositol.

### 3.5. Metabolic pathway analysis

**Figure 8.**
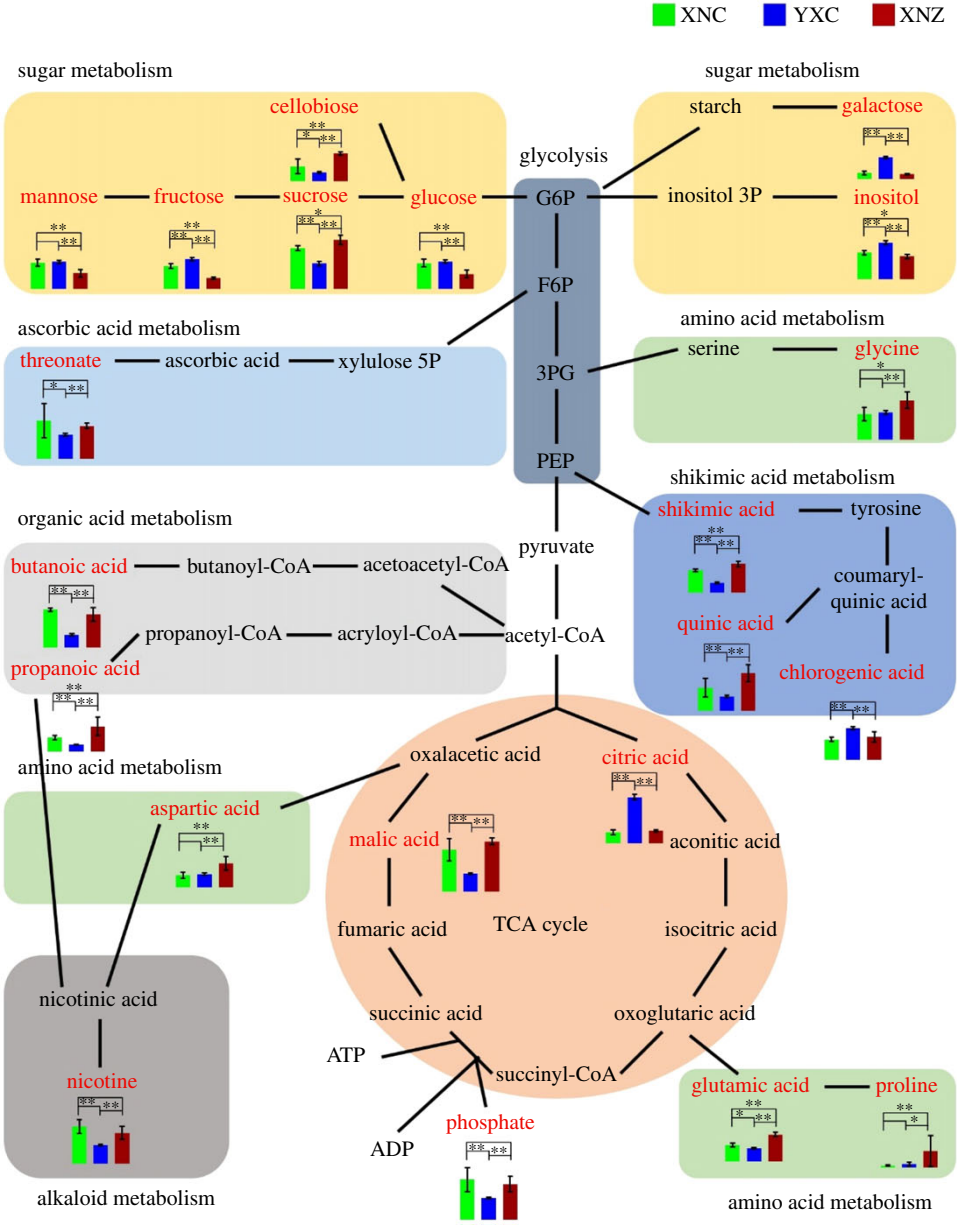
Metabolic pathway map of the different metabolites. Green, blue and red columns indicate the relative concentration of metabolites in XNC, YXC and XNZ, respectively. Asterisks * and ** indicate a significant difference between two samples at the 0.05 and 0.01 probability level, respectively. The metabolites are abbreviated as follows: G6P, glucose-6-phosphate; F6P, fructose-6-phosphate; 3PG, 3-phosphoglycerate; PEP, phosphoenolpyruvic acid; inositol 3P, inositol-3-phosphate; xylulose 5P, xylulose-5-phosphate, ATP, adenosine triphosphate; ADP, adenosine diphosphate.

To deeply understand the differences in the metabolic networks among three samples, all the differential metabolites were submitted to the KEGG website for metabolic pathway enrichment analysis. The results showed that 21 of the differential metabolites were located in various pathways, whereas the other three carbohydrate compounds (lactulose, mannopyranose, 2-*O*-glycerol-galactopyranoside) were not found in any pathway. A generalization of differential metabolites among three groups of the tobacco leaf samples is seen in a metabolic pathway in figure 8. Five primary metabolic pathways (glycolysis, sugar metabolism, amino acid metabolism, tricarboxylic acid cycle (TCA) and organic acid metabolism), and three secondary metabolic pathways (ascorbic acid metabolism, shikimic acid metabolism and alkaloid metabolism) were extracted and linked based on the KEGG pathway database. The above involved metabolic pathways were mostly similar to that observed in flue-cured tobacco among different planting regions and climate factors [2].

### 3.6. Correlation analysis

**Figure 9.**
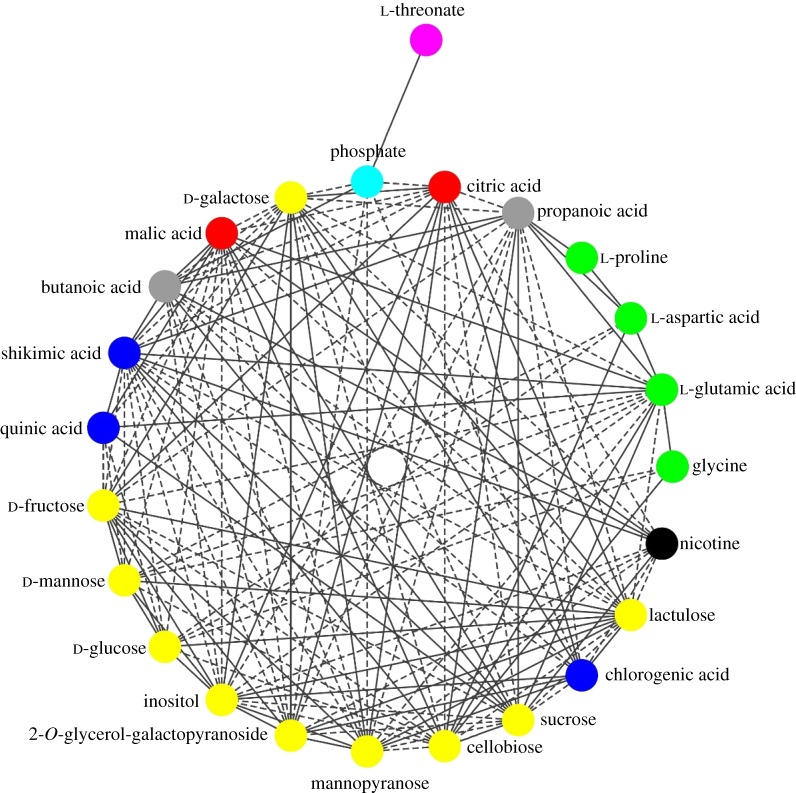
Correlation analysis between the identified differential metabolites. The dashed lines between metabolites represent negative correlation, whereas solid lines represent positive correlation. All correlations in the figure reflect Pearson correlation coefficient values above the threshold (*p* > 0.65).

To investigate the metabolite–metabolite correlation of the differential metabolites, the Pearson correlation coefficient values were filtered by the threshold (*p *> 0.65), and a network analysis was constructed with 24 differential metabolites and 146 edges (figure 9). The correlation of the differential metabolites indicated that 77 were negative and 69 were positive. Most of the amino acids and organic acids in the differential metabolites were positively correlated with each other, but negatively correlated with the carbohydrates. Citric acid and two disaccharides (cellobiose and sucrose) were exceptions. The largest number of correlations was 18, and were related to shikimic acid, whereas the fewest correlations were related to threonate, which only correlated with phosphate. The correlation details among differential metabolites are shown in electronic supplementary material, table S3.

## 4. Discussion

GC–MS has been used widely as a metabolomics method to investigate the metabolic profiles of plant materials and to identify the significantly differential metabolites between samples [4,5]. In the current study, significant differences in the metabolic profiles between two important Chinese cultivars of tobacco leaves grown in two locations were observed using a pseudo-targeted GC–MS SIM method, and 24 differential metabolites were excavated and confirmed by multivariate analysis. Metabolic pathway enrichment and correlation analysis of differential metabolites were also conducted.

The stability and accuracy of detection methods are the prerequisites for a successful experiment. It is difficult to obtain too many standard compounds in metabolic profiling analysis [25]. To solve the problem, QC samples were generally used to evaluate the stability and repeatability of the whole analytical process [1,12]. In the current study, all of QC samples were clustered together and located at the centre of the PCA score plot (figure 3*b*), and most of the peaks had an RSD value less than 30% and represented almost all of the total responses (figure 2), which is consistent with the results of previous research [12,24]. PLS-DA is a supervised method that is frequently used in multivariate analysis. The results of permutation test showed that the R2-intercept was 0.249 and the Q2-intercept was −0.327, indicating that our model was properly fitted. Based on the results, it was shown that the reproducibility and precision of this method were acceptable for the metabolomics analysis of tobacco leaves.

Xuanen County was selected as the growing site to investigate the metabolic profile of tobacco leaves from Hubei Province, which is an important planting area of middle flavour flue-cured tobacco [13]. Yuxi City in Yunnan Province, which is the largest tobacco planting region in China, was also selected. In the present study, 13 differential metabolites were observed between tobacco leaves from Xuanen County and those from Yuxi City, including seven organic and fatty acids (malic acid, citric acid, shikimic acid, quinic acid, phosphate, butanoic acid and threonate), four carbohydrates (galactose, 2-*O*-glycerol-galactopyranoside, mannopyranose and lactulose), and two secondary metabolites (nicotine and chlorogenic acid) (figures 6 and 7). Among them, the levels of differential acids (except for citric acid) in tobacco leaves from Xuanen County were significantly higher than in tobacco leaves from Yuxi City, whereas the carbohydrates levels were lower. Similar distributed trends of differential metabolites were reported between tobacco leaves from Yunnan and Guizhou in previous studies [1,2]. It has been reported that sufficient sun exposure time and higher altitude can enhance sugar content [1,2]. The total sun exposure time is shorter and the altitude is lower in Xuanen County than in Yuxi City, which might be the probable cause of our results from the two regions. The tobacco leaves from Hubei and Guizhou are classified as middle flavour type [13,19], but the levels of citric acid, which is an important metabolite in the TCA cycle, were different between the two regions. This might be due to the differences in environmental conditions between the two regions and the tobacco flavour complexity. Nicotine and chlorogenic acid, which are closely related to climatic factors, are two important secondary metabolites of tobacco leaves, and contribute greatly to tobacco flavour and quality [1,20,26,27]. Nicotine stimulation plays an important role in the physiological effects of smoking [1,27]. Numerous studies reported that the nicotine content of tobacco leaves could increase with the increased sun exposure time, rainfall, nitrogen fertilizer application amount, and so on [28]. In this study, the nicotine level in the tobacco leaves from Xuanen County was lower than the tobacco from Yuxi City, which is consistent with the previous report [29]. However, no positive correlation was found between nicotine content and sun exposure time in our study. The reason for this discrepancy might be that the accumulation of nicotine is determined by a combination of many factors, such as climate, soil, cultivar and cultivation techniques, rather than one factor [1,28]. Chlorogenic acid is the main phenolic in tobacco leaves, and contributes to mild, sweet flavour and the scent of baking in tobacco products [26]. The enhancement of chlorogenic acid was induced by increased altitude and long sun exposure time, which is consistent with our results, and might be related to near-ultraviolet light and the intensity of visible light [30,31]. Taking into account the different environments of the two regions, the higher levels of nicotine and lower levels of chlorogenic acid in the tobacco from Xuanen County than the tobacco from Yuxi City contributed to the flavour characteristics of each.

In the present study, six differential tobacco leaf metabolites were observed between Cuibi-1 and Zhongyan-100, including four amino acids (glycine, glutamic acid, aspartic acid and proline) and two monosaccharides (glucose and mannose) (figures 6 and 7). In addition, the levels of amino acids in Zhongyan-100 were significantly higher than in Cuibi-1, whereas the monosaccharide levels were lower. Among them, proline, which synthesized from glutamic acid, is a proteinogenic amino acid and is essential for primary metabolism. Numerous studies have reported that proline accumulation was found in plants under different environmental stresses, and that proline can act as a signalling molecule to participate in plant resistance [32–34]. Thus, high levels of amino acids in Zhongyan-100 could partially contribute to the high resistance of this cultivar. As an important monosaccharide, glucose has fundamental and multiple effects on plant metabolism, and is reported to modulate biosynthesis of plant secondary metabolites that are involved in the quality and flavour of tobacco leaves [35]. Therefore, high levels of monosaccharides in Cuibi-1, especially glucose, could explain the high aroma of the cultivar. In addition, the levels of propanoic acid and four carbohydrates (sucrose, fructose, cellobiose and inositol) were significantly different between the intersection of three samples, indicating that these differential metabolites were simultaneously influenced by both geographical origin and cultivar.

The flavour and quality of plant edible organs were principally governed by the levels and ratios of sugar and organic acid [36,37]. Sugar and organic acid catabolism as the primary metabolism in the plants were also involved in the biosynthesis of secondary metabolites, including amino acids, vitamins and aroma volatiles, which then further influence the flavour and quality [35,37]. The results of correlation analysis in our study showed that most of the differential carbohydrates were negatively correlated with the differential amino acids and organic acids (figure 9). This is probably related to the regulation of development and metabolism of tobacco leaves. Moreover, citric acid, sucrose and cellobiose were found as exceptions for each class, but the reason for this was not clear and still needs to be investigated in the future.

## 5. Conclusion

The metabolic profiling of Cuibi-1 and Zhongyan-100 flue-cured tobacco middle leaves in different planting regions was analysed using GC–MS with the pseudo-targeted SIM method. Twenty-four differential metabolites were identified via multivariate analysis, including 13 geographical region-related metabolites (malic acid, citric acid, shikimic acid, quinic acid, phosphate, butanoic acid, threonate, galactose, 2-*O*-glycerol-galactopyranoside, mannopyranose, lactulose, nicotine and chlorogenic acid), six cultivar-related metabolites (glycine, glutamic acid, aspartic acid, proline, glucose and mannose) and five metabolites (sucrose, fructose, cellobiose, inositol and propanoic acid) that are involved in both. The results highlight the effect of environment and cultivar on tobacco leaf metabolism.

## Supplementary Material

Supplementary Tables

## Data accessibility

All data used in this article are present in the article and its electronic supplementary material.

## References

[1] Zhang L, Wang XY, Guo JZ, Xia QL, Zhao G, Zhou HN, Xie FW 2013 Metabolic profiling of Chinese tobacco leaf of different geographical origins by GC-MS. J. Agric. Food Chem. 61, 2597–2605. (doi:10.1021/jf400428t) 23441877 10.1021/jf400428t

[2] Zhao YN et al. 2013 Investigation of the relationship between the metabolic profile of tobacco leaves in different planting regions and climate factors using a pseudotargeted method based on gas chromatography/mass spectrometry. J. Proteome Res. 12, 5072–5083. (doi:10.1021/pr400799a) 24090132 10.1021/pr400799a

[3] Fu XM, Zhou Y, Zeng LT, Dong F, Mei X, Liao YY, Watanabe N, Yang ZY 2017 Analytical method for metabolites involved in biosynthesis of plant volatile compounds. RSC Adv. 7, 19363 (doi:10.1039/C7RA00766C)

[4] Duan LX et al. 2012 Use of the metabolomics approach to characterize Chinese medicinal material Huangqi. Mol. Plant 5, 376–386. (doi:10.1093/mp/ssr093) 22138859 10.1093/mp/ssr093

[5] Agarrwal R, Bentur JS, Nair S 2014 Gas chromatography mass spectrometry based metabolic profiling reveals biomarkers involved in rice-gall midge interactions. J. Integr. Plant Biol. 56, 837–848. (doi:10.1111/jipb.12244) 25059749 10.1111/jipb.12244

[6] Qu GR, Quan S, Mondol P, Xu J, Zhang DB, Shi JX 2014 Comparative metabolomics analysis of wild type and *mads3* mutant rice anthers. J. Integr. Plant Biol. 56, 849–863. (doi:10.1111/jipb.12245) 25073727 10.1111/jipb.12245

[7] Clarke JD, Alexander DC, Ward DP, Ryals JA, Mitchell MW, Wulff JE, Guo LN 2013 Assessment of genetically modified soybean in relation to natural variation in the soybean seed metabolome. Sci. Rep. 3, 3082 (doi:10.1038/srep03082) 24170158 10.1038/srep03082 PMC3812653

[8] Lin H, Rao J, Shi JX, Hu CY, Cheng F, Wilson ZA, Zhang DB, Quan S 2014 Seed metabolomics study reveals significant metabolite variations and correlations among different soybean cultivars. J. Integr. Plant Biol. 56, 826–836. (doi:10.1111/jipb.12228) 24942044 10.1111/jipb.12228

[9] Yang Y, Zhao XJ, Pan Y, Zhou ZQ 2015 Identification of the chemical compositions of Ponkan peel by ultra performance liquid chromatography coupled with quadrupole time-of-flight mass spectrometry. Anal. Methods 8, 893–903. (doi:10.1039/C5AY02633D)

[10] Liu XG, Lu X, Wang JX, Wu B, Lin L, Wang HY, Guo RZ, Li P, Yang H 2017 Combining paired analytical metabolomics and common garden trial to study the metabolism and gene variation of *Ginkgo biloba* L. cultivated varieties. RSC Adv. 7, 55309 (doi:10.1039/C7RA06229J)

[11] Li LL, Zhao JY, Zhao YN, Lu X, Zhou ZH, Zhao CX, Xu GW 2016 Comprehensive investigation of tobacco leaves during natural early senescence via multi-platform metabolomics analyses. Sci. Rep. 6, 37976 (doi:10.1038/srep37976) 27897248 10.1038/srep37976 PMC5126694

[12] Zhao YN, Zhao CX, Li YL, Chang YW, Zhang JJ, Zeng ZD, Lu X, Xu GW 2014 Study of metabolite differences of flue-cured tobacco from different regions using a pseudotargeted gas chromatography with mass spectrometry selected-ion monitoring method. J. Sep. Sci. 37, 2177–2184. (doi:10.1002/jssc.201400097) 24865655 10.1002/jssc.201400097

[13] Tang YJ 2011 On aroma type of flue-cured tobacco. Chin. Tobacco Sci. 32, 1–7.

[14] Zhao JY et al. 2014 Study of polar metabolites in tobacco from different geographical origins by using capillary electrophoresis-mass spectrometry. Metabolomics 10, 805–815. (doi:10.1007/s11306-014-0631-4)

[15] Li LL, Lu X, Zhao JY, Zhang JJ, Zhao YN, Zhao CX, Xu GW 2015 Lipidome and metabolome analysis of fresh tobacco leaves in different geographical regions using liquid chromatography-mass spectrometry. Anal. Bioanal. Chem. 407, 5009–5020. (doi:10.1007/s00216-015-8522-8) 25701418 10.1007/s00216-015-8522-8

[16] Zhao JY et al. 2016 Metabolic profiling with gas chromatography-mass spectrometry and capillary electrophoresis-mass spectrometry reveals the carbon-nitrogen status of tobacco leaves across different planting areas. J. Proteome Res. 15, 468–476. (doi:10.1021/acs.jproteome.5b00807) 26784525 10.1021/acs.jproteome.5b00807

[17] Sun B, Xue SL, Zhang F, Luo ZP, Wu MZ, Chen Q, Tang HR, Lin FC, Yang J 2015 A quantitative real-time PCR-based strategy for molecular evaluation of nicotine conversion in burley tobacco. Int. J. Mol. Sci. 16, 27 422–27 432. (doi:10.3390/ijms161126038) 10.3390/ijms161126038 PMC4661896 26593897

[18] Zhao MQ, Chen QH, Zhao MS, Hu HX, Cheng YY, Xu CK, Liu GS 2008 Effects of ecological condition in Nanyang on chemical components and aroma substances in different genotypic flue-cured tobacco leaves. Acta Tabacaria Sinica 14, 37–41.

[19] Sun B et al. 2013 Effects of different environmental locations on alkaloid accumulation in tobacco leaves in China. Food Agric. Environ. 11, 1337–1342.

[20] Sun B, Zhang F, Zhou GJ, Chu GH, Huang FF, Wang QM, Jin LF, Lin FC, Yang J 2013 Genetic variation in alkaloid accumulation in leaves of *Nicotiana*. J. Zhejiang Univ. SCI. B. 14, 1100–1109. (doi:10.1631/jzus.B1300130) 24302710 10.1631/jzus.B1300130 PMC3863368

[21] Xu QH, Lv DS, Zhang YH, Li W, Yuan TJ, Yang SH 2017 Identification of flue-cured tobacco flavor style using GC/MS fingerprint of aroma component extracted by dichloromethane. Tob. Sci. Technol. 50, 30–40.

[22] Lin CX, Zhang XQ 2015 Effects of harvest way and flue-curing practice on quality of tobacco cv. ‘Cuibi No. 1’. Fujian Agric. Sci. Technol. 5, 73–77.

[23] Jia XH, Wang YY, Tong DR, Feng QF, Luo CG, Chen ZQ, Fu XK, Liu SY, Wang SM 2006 Development of a new flue-cured tobacco variety Zhongyan-100 (CF965) and its application evaluation. Acta Tabacaria Sinica 12, 20–25.

[24] Li Y, Pang T, Wang XL, Li QH, Lu X, Xu GW 2013 Gas chromatography-mass spectrometric method for metabolic profiling of tobacco leaves. J. Sep. Sci. 36, 1545–1552. (doi:10.1002/jssc.201201037) 21560246 10.1002/jssc.201100106

[25] Li Y, Pang T, Li YL, Ye GZ, Lu X, Xu GW 2011 Chemical properties investigation of commercial cigarettes by a ‘pseudo’ targeted method using GC-MS-selected ions monitoring. J. Sep. Sci. 34, 1447–1454. (doi:10.1002/jssc.201100106) 23436432 10.1002/jssc.201201037

[26] Wang H, Zhao M, Yang B, Jiang Y, Rao G 2008 Identification of polyphenols in tobacco leaf and their antioxidant and antimicrobial activities. Food Chem. 107, 1399–1406. (doi:10.1016/j.foodchem.2007.09.068)

[27] Sears MT, Zhang HB, Rushton PJ, Wu M, Han SC, Spano AJ, Timko MP 2014 NtERF32: a non-NIC2 locus AP2/ERF transcription factor required in jasmonate-inducible nicotine biosynthesis in tobacco. Plant Mol. Biol. 84, 49–66. (doi:10.1007/s11103-013-0116-2) 23934400 10.1007/s11103-013-0116-2

[28] Dai M 2000 Relationship between climate factors and leaf chemical composition in some tobacco leaf production areas in China. Acta Tabacaria Sinica 6, 27–34.

[29] Sun JG, He JW, Wu FG, Tu SX, Yan TJ, Si H, Xie H 2011 Comparative analysis on chemical components and sensory quality of aging flue-cured tobacco from four main tobacco areas of China. Agr. Sci. China 10, 1222–1231. (doi:10.1016/S1671-2927(11)60113-2)

[30] Tso TC, Kasperbauer MJ, Sorokin TP 1970 Effect of photoperiod and end-of-day light quality on alkaloids and phenolic compounds of tobacco. Plant Physiol. 45, 330–333. (doi:10.1104/pp.45.3.330) 5423471 10.1104/pp.45.3.330 PMC396407

[31] Anderson R, Kasperbauer MJ 1973 Chemical composition of tobacco leaves altered by near-ultraviolet and intensity of visible light. Plant Physiol. 51, 723–726. (doi:10.1104/pp.51.4.723) 16658399 10.1104/pp.51.4.723 PMC366335

[32] Szabados L, Savouré A 2009 Proline: a multifunctional amino acid. Trends Plant Sci. 15, 89–97. (doi:10.1016/j.tplants.2009.11.009) 20036181 10.1016/j.tplants.2009.11.009

[33] Székely G et al. 2008 Duplicated *P5CS* genes of Arabidopsis play distinct roles in stress regulation and developmental control of proline biosynthesis. Plant J. 53, 11–28. (doi:10.1111/j.1365-313X.2007.03318.x) 17971042 10.1111/j.1365-313X.2007.03318.x

[34] Suo JW, Zhao Q, David L, Chen SX, Dai SJ 2017 Salinity response in chloroplasts: insights from gene characterization. Int. J. Mol. Sci. 18, 1011 (doi:10.3390/ijms18051011) 10.3390/ijms18051011 PMC5454924 28481319

[35] Miao HY et al. 2016 Glucose enhances indolic glucosinolate biosynthesis without reducing primary sulfur assimilation. Sci. Rep. 6, 31854 (doi:10.1038/srep31854) 27549907 10.1038/srep31854 PMC4994012

[36] Kader AA 2008 Flavor quality of fruits and vegetables. J. Sci. Food Agric. 88, 1863–1868. (doi:10.1002/jsfa.3293)

[37] Chen M, Jiang Q, Yin XR, Lin Q, Chen JY, Allan AC, Xu CJ, Chen KS 2012 Effect of hot air treatment on organic acid- and sugar-metabolism in Ponkan (*Citrus reticulata*) fruit. Sci. Hortic. 147, 118–125. (doi:10.1016/j.scienta.2012.09.011)

